# Nasal Myiasis: A Case Report

**Published:** 2018-09

**Authors:** Shokrollah SALMANZADEH, Mahmoud RAHDAR, Sharif MARAGHI, Fatemeh MANIAVI

**Affiliations:** 1. Infectious and Tropical Diseases Research Center, Ahvaz Jndishapur University of Medical Sciences, Ahvaz, Iran; 2. Dept. of Medical Mycoparasitology, Ahvaz Jundishapur University of Medical Sciences, Ahvaz, Iran; 3. Health Research Institute, Thalassemia, and Hemoglobinopathy Research Center, Ahvaz Jundishapur University of Medical Sciences, Ahvaz, Iran; 4. Shafa Hospital, Ahvaz Jndishapur University of Medical Sciences, Ahvaz, Iran

**Keywords:** Nasal myiasis, Hospital, Iran

## Abstract

Myiasis is caused by invasion of larvae stage of dipterans fly in living tissue of vertebrate host including human and animals. The most important family is Calliphoridae and included *Calliphora, Lucilia, Chrysomyia* and *Cochliomyia* genus. A 35-yr-old man with gastric cancer history referred to Golestan Hospital in Ahvaz Southwest of Iran in 2015. He was infected by nasal myiasis from *Lucilia* spp. in ICU. The genus of third larvae stage was identified by microscopic examination and culture of pupa. The population of flies inducing myiasis should be controlled in hospitals.

## Introduction

For the first time, myiasis term was introduced in 1940 (1). He used the myiasis term for invasion of eggs or larvae stage of dipterans flies to tissue and body cavity of the live vertebrate host. Several dipterans species can cause obligatory or facultative myiasis in vertebrate host such as animals and human. The most important family for causing myiasis is Calliphoridae family which included *Calliphora, Lucilia, Chrysomyia* and *Cochliomyia* genera.

The larvae or eggs are deposed on body cavity or injured skin and then penetrate to live tissue. They spend different time on their host according to species and feed on dead or living tissue and fluid. After several days, they change to pupa stage and finally leave their hosts.

The distribution of the disease is worldwide and the prevalence was reported in many countries especially in tropical area with high humidity which is suitable for fly life cycle (2). The most common infested location is opening orifice such as nose, eyes, ears, anus, vagina and injured skin of their host. Oral myiasis is rarer compared to other infestation forms due to less exposure to environment and usually occurs in debilitated patients with comma history (3).

This study reports a nasal myiasis case in a patient with neurological deficit.

## Case Report

The patient was a 35-yr-old man with gastric cancer history for one year referred to Golestan Hospital, Ahvaz southwest of Iran in 2015 due to clinical signs including vomiting, loss of consciousness, food intolerances, impotence, icterus and paleness for 1 year. He had undergone chemotherapy bytaxotel (50mg), cisplatin (35mg) and flucytosine (500mg) according to his practitioner recommendation for four days. The chemotherapy schedule was repeated 4 times. The vital sign and biochemical parameters were: BP: 100/60 mm/Hg, PR: 110/min, RR= 28 /min, OT: 38.5C. The other biochemical parameters were:

ALT = 166, AST= 250, WBC: 23.80x 10 3, HB: 7.4, PLT: 56x10 3, FBS: 93, Na: 130, K: 5.9, Ca: 8.1, P: 3.9, bilirubin (T): 33.6, bilirubin (D): 30.1BUN= 149, Cr= 5.3 and in urinalysis: PRO 2+, Hb: 3+.

**Fig. 1:**
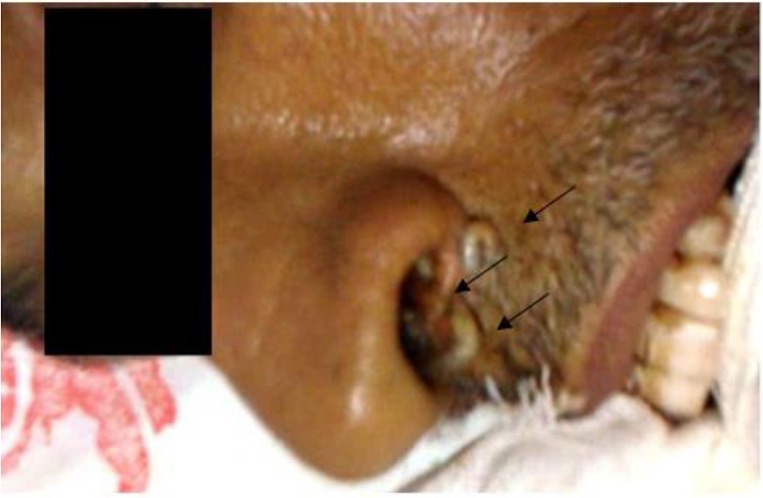
*Lucilia* larvae expelled from nasal cavity of comatose patient (Original)

**Fig. 2:**
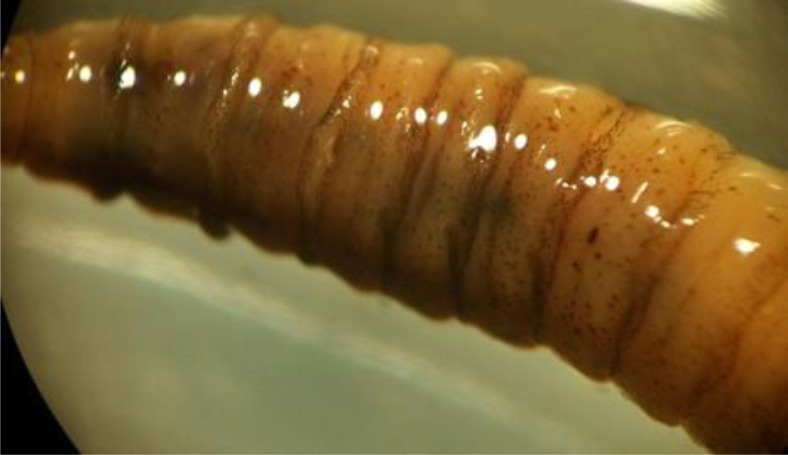
Larva (third stage) of *Luilia* spp. removed from nasal cavity (Original)

**Fig. 3:**
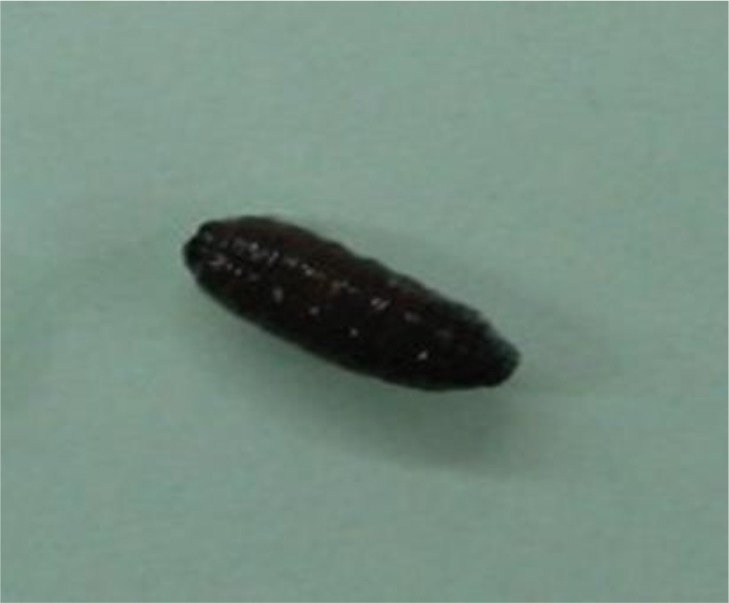
Pupa stage of *Lucilia* spp. in nasal cavity (Original)

The patient was transferred to ICU due to respiratory distress. After few days, several larvae and pupa stage were seen in nasal and oral cavity (Fig. 1). The larvae and pupa were removed by forceps and transferred to parasitology department for precise diagnosis (Fig. 2, 3). Nasal myiasis was recognized by infectious department consulting.

**Fig. 4:**
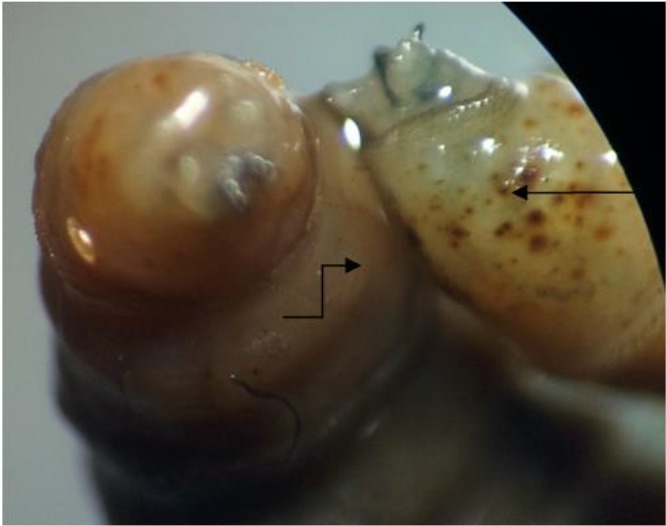
Anterior spiracle from *Lucilia* spp. removed from nasal cavity (Original)

**Fig. 5:**
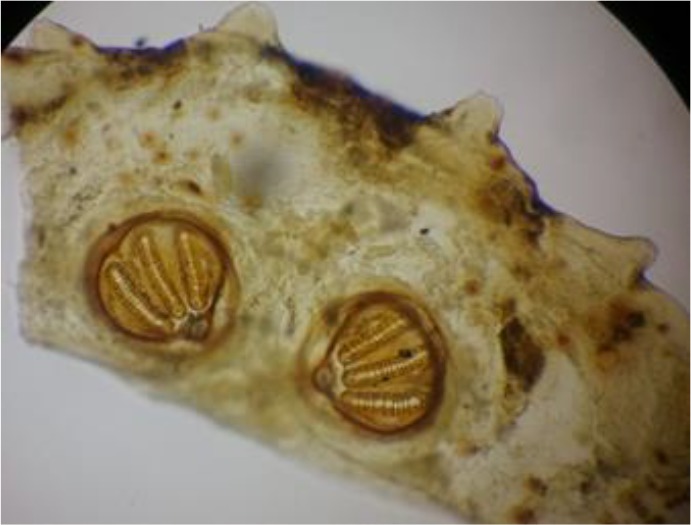
Posterior spiracle from *Lucilia* spp. removed from nasal cavity (Original)

**Fig. 6:**
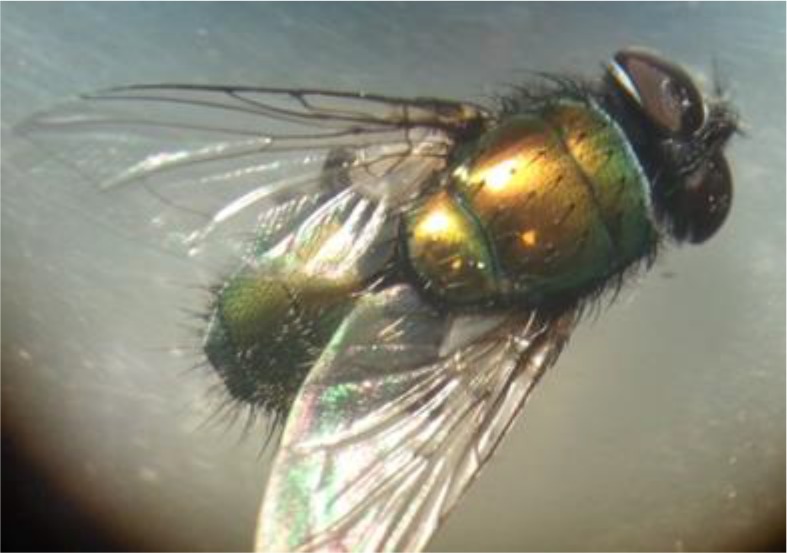
Adult fly emerged from pupa stage (Original)

The patient was treated with turpentine, meropenem, vancomycin, and fluconazole for myiasis, pneumonia and candidiasis infection. The patient died after 5 d. The identification of third stages of larvae was performed according to internal and external morphological characteristics. Anterior and posterior air spiracles were removed from the body of maggots and examined microscopically (Fig. 4–5). The pupa was cultured in room temperature for recognizing adult fly (Fig. 6). According to larvae, pupa and adult characters*, Lucilia* spp. was identified (4).

## Discussion

Myiasis is caused by invasion of larvae stage of dipterans fly in living tissue of vertebrate host. The disease classifies into two type forms according to parasite biology included facultative myiasis and obligatory myiasis (5).

The facultative myiasis is caused by invasion of larva stage to livingor necrotic tissue (carcass). *Calliphora, Lucilia*, and *Phormia* are common genera which cause facultative myiasis.

Obligatory myiasis related to flies that need to deposit their larva or eggs on living tissue of host for completing their cycle. These flies especially belonged to Calliphoridaefamilies such as *Cochliomyia*, *Chrysomia* and *Cordylobia* species. The larva can penetrate to normal skin and natural orifice. The other classification of myiasis is performed by involved organ. In this point of view, oral myiasis, gastrointestinal myiasis, urogenital myiasis has been considered.

Adults’ flies are attracted to animals via urine, feces and respiratory odor and laid their eggs or first stage larva on natural opening or injured skin.

After deposition of larva on body, the larva penetrated to tissue by a chitinous apartment called cephalopharyngeal skeleton apparatus located in anterior end of the larva. Secretion of proteolysis enzymes and cephalopharyngeal skeleton movement help the larva to penetrate and feed on damaged tissue and cause inflammation. Secondary infections by pathogen bacteria commonly are found in myiasis ulceration and led to death due to septicemia outcome in untreated cases (3). The incidence of oral myiasis compared to another clinical myiasis, is rare because of the oral cavity are not permanently exposed to invasion of flies. Myiasis infestation commonly occurred in poor hygienic condition and warm and rainy seasons (6). The most risk factors for acquisition of the infection are poor hygienic condition of mentally patients (7), alcohol-dependent consumers, epileptic patients with face injured following seizures attack, very poor dental hygienic persons (5) neurological disability in-patients with coma history (4, 8) and surgery (9). In children, predisposing factors are lips and mouth figuration disorders, thumb sucking habits and continuous mouth breathing (10).

*L. serrate* can cause nasal myiasis in patient with dyspnea disorder. The patient was referred to hospital for treatment of dyspnea. The third stage of larva was removed from nasal and after morphological study, *L.sericata* were identified. *Lucilia* has metallic color (green blowflies) and it considered as facultative myiasis producer (11). They have worldwide in distribution and generally were seen around meat or animal carcasses. They also present indoor in human habitats and attracted to hospitals because of patient odor and lay their eggs on natural opening of patients with comatose condition. *Phormia regina* also can produce oral myiasis in hospital (12).

There are many myiasis reports of dipteral flies from Iran. An oral myiasis was presented in a 3-yr rural boy. He was infected by *O. ovis* (Sheep nasal botfly cespo) in mouth cavity with sever gingivitis. The third stage of *O. ovis* were removed from periodontal tissue (13, 14). A case study of nasal myiasis was reported caused by C. bezziana from a 74 old woman in Gonabad. She referred to hospital due to respiratory disorder, dyspnea and fever. She had contacted with domestic animals and history of chronic obstructive pulmonary disease. The treatment of antibiotics was not successful. After some day’s observation in ICU, numerous larvae were detected from her nostrils. The larvae were transfered to entomology department for diagnosis and C. bezzianawas diagnosed according to morphological entomology characters (15).

C. bezziana is one of the important species to cause myiasis in human and animal population. The invasion of adult fly is acquired in wounds and normal body orifice during spring and summer. A myiasis case report was presented in scalp of a 5 year old boy with severe headache and precise observation revealed infected by C. bezziana-larvae (16).

Nasal myiasis was reported in a 45 yr old man in Iranshahr (Sistan & Balochistan Province) of Iran. The larvae were removed from nasal and final examination showed that are belong to *C. bezziana* species (17). Urogenital myiasis caused by *Chrysomia bezziana* in 36 yr old women with abdominal pain and dysuria from Isfahan (18).

In addition to surgery removal of the maggots, some drugs therapy is needed. The posterior spiracles of larva usually come out from lesion for breathing. Therefore, occlusion of wounds by paraffin, mineral oil, animal fat and beeswax inhibited oxygen accessibility to larva (19) and appropriate antibiotics should be used to treat bacterial infection.

## Conclusion

Blowflies (Diptera, Calliphoridae) can be presented in area with poor hygienic condition and they are very harmful to patients with some disability especially who hospitalized in ICU and CCU unit. Therefore in these places, population of myiasis flies should be controlled by using insecticide, cleaning and covering the wounds and performing good sanitation in hospitals.

## Ethical considerations

Ethical issues (Including plagiarism, informed consent, misconduct, data fabrication and/or falsification, double publication and/or submission, redundancy, etc.) have been completely observed by the authors.

## References

[1] Hope FW (1840). On insects and their larvae occasionally foundin human body. Transactions of the Entomological Society of London, 2:256–71.

[2] Fabio Francesconi F Lupi O (2012). Myiasis. Clin Microbiol Rev, 25(1):79. 22232372 10.1128/CMR.00010-11 PMC3255963

[3] Moshref M Ansari G Lotfi A (2008). Oral gingival myiasis: a case report. Int J Trop Med Publ Health, 3:97–100.

[4] Zachariah JE Sehgal K Dixit UB (2014). Oral myiasis: a case report. Spec Care Dentist, 34(1):51–3. 24382372 10.1111/scd.12016

[5] Raffaldi I Scolfaro C Pinon M (2013). A strange gingival swelling in an Italian child: a case of oral myiasis. Infez Med, 21(1):56–9. 23524903

[6] Bhola N Jadhav A Borle R (2012). Primary oral myiasis: a case report. Case Rep Dent, 2012:734234. 23125939 10.1155/2012/734234 PMC3485497

[7] Kumar P Singh V (2012). Oral myiasis: case report and review of literature. Oral Maxillofac Surg, 18(1):25–9. 23179955 10.1007/s10006-012-0373-2

[8] Nazni WA Jeffery J Lee HL (2011). Nosocomial nasal myiasis in an intensive care unit. Malays J Pathol, 33(1):53–6. 21874753

[9] Jan TA Redjal N Walcott BP (2013). Intranasal myiasis: A rare complication of transnasal skull base surgery. J Clin Neurosci, 20(8):1178–80. 23669173 10.1016/j.jocn.2012.09.028

[10] De Souza Barbosa T Salvitti Sá Rocha RA Guirado CG (2008). Oral infection by Diptera larvae in children: a case report. Int J Dermatol, 47:696–9. 18613876 10.1111/j.1365-4632.2008.03725.x

[11] Youssefi M Rahimi M Marhaba Z (2012). Occurrence of Nasal Nosocomial Myiasis by Lucilia sericata (Diptera: Calliphoridae) In North of Iran. Iran J Parasitol, 7(1):104–8. PMC3488829 23133480

[12] Vazirianzadeh B Rahdar M Maraghi S (2008). Phormia regina (Diptera, Cyclorrhapha: Caliphoridae): Myaisis agents, status and principals of prevention in khoozestan SW Iran. J Exp Zool India, 11(2):473–6.

[13] Hakimi R Yazdi I Oral Mucosa Myiasis Caused By *Oestrus ovis*. Arch Iran Med http://www.ams.ac.ir/AIM/0253/0253194.htm

[14] Hazratian T Tagizadeh A Chaichi M (2017). Pharyngeal Myiasis Caused by Sheep Botfly, *Oestrus ovis* (Diptera: Oestridae). J Arthropod Borne Dis, 11(1): 166–170. 29018832 PMC5629300

[15] Mircheraghi SF Mircheraghi SF Ramezani Awal Riabi H (2016). Nasal Nosocomial Myiasis Infection Caused by Chrysomya bezziana (Diptera: Calliphoridae) Following the Septicemia: A Case Report. Iranian Journal of Parasitology, 11(2): 284–289. 28096867 PMC5236110

[16] Soleimani Ahmadi M Nasirian H Nazemi Gheshmi AM (2009). Human Extensive Head Skin Myiasis. Iranian Journal of Public Health, 38(1): 134–138.

[17] Tirgari S Nateghpour M Jahanian AH (2003). Case report: First Record of Human Myiasis caused by *Chrysomia bezziana* (Villeneuve) in Iran (Diptera, Calliphoridae). Iranian J Publ Health, 32 (3): 68–70.

[18] Jdalayer T Maleki M Moghtaderi M (1978). Human urogenital myaiasis caused by *Chrysomyia bezziana*. Iran J Public Health, 7(3): 116–117.

[19] McGraw TA Turiansky GW (2008). Cutaneous myiasis. J Am Acad Dermatol, 58(6):907–26. 18485982 10.1016/j.jaad.2008.03.014

